# Phasic dopamine release in the rat nucleus accumbens predicts approach and avoidance performance

**DOI:** 10.1038/ncomms13154

**Published:** 2016-10-27

**Authors:** Ronny N. Gentry, Brian Lee, Matthew R. Roesch

**Affiliations:** 1 Department of Psychology, University of Maryland, College Park, Maryland 20742, USA; 2 Program in Neuroscience and Cognitive Sciences, University of Maryland, College Park, Maryland 20742, USA

## Abstract

Dopamine (DA) is critical for reward processing, but significantly less is known about its role in punishment avoidance. Using a combined approach-avoidance task, we measured phasic DA release in the nucleus accumbens (NAc) of rats during presentation of cues that predicted reward, punishment or neutral outcomes and investigated individual differences based on avoidance performance. Here we show that DA release within a single microenvironment is higher for reward and avoidance cues compared with neutral cues and positively correlated with poor avoidance behaviour. We found that DA release delineates trial-type during sessions with good avoidance but is non-selective during poor avoidance, with high release correlating with poor performance. These data demonstrate that phasic DA is released during cued approach and avoidance within the same microenvironment and abnormal processing of value signals is correlated with poor performance.

## Abstract (web summary)

Reward seeking behaviors involve dopamine (DA) release but the circuits underlying avoidance behavior remain comparatively understudied. Here the authors show that phasic increases in DA release in rats are higher for reward and avoidance cues compared with neutral cues and are positively correlated with poor avoidance.

While a breadth of literature has examined the role of phasic dopamine (DA) release within the context of unexpected rewards and the cues that come to predict them^1^,^2^, fewer studies have explored the function of DA signalling in aversive situations. Both reward-seeking and punishment-avoidance paradigms promote instrumental responding^3^,^4^,^5^, but these behaviours are differentially governed by positive and negative reinforcement learning strategies, respectively. It is still unknown how conditioned stimuli promote avoidance behaviours, how these behaviours are modified by DA release, or if these effects are analogous to those seen during appetitive tasks. These questions have spurred discussion regarding the further heterogeneity of the dopamine response and a recent surge of models aiming to describe negative reinforcement using DA-like prediction error signalling^3^,^4^,^6^,^7^,^8^,^9^. To date, these issues have not been adequately addressed, largely because few studies have examined DA signals in the context of both positive and negative reinforcement.

Since the mechanisms governing punishment avoidance have been studied considerably less than those of reward seeking, the circuit underlying avoidance behaviour remains poorly understood. The behavioural processes that guide punishment avoidance are complex, involving both an initial Pavlovian response and a secondary instrumental component^10^,^11^,^12^,^13^. Cues that predict the possibility of shock also produce fear, often leading to freezing behaviour and inaction that reduces the likelihood of avoidance^11^,^14^,^15^. The transition from freezing to successfully pressing a lever or shuttling to avoid foot shock requires overcoming this initial fear response to initiate action^14^,^16^. This is very different from behaviour driven by reward; cues that predict reward generally arouse animals, promoting action and increasing the probability of responding^1^,^3^,^5^,^17^.

Given these complications, it is no surprise that punishment avoidance tasks are generally more difficult to learn than reward-seeking tasks. This distinction is greatly influenced by the mode of response (nose poke, lever press and shuttle response) employed within the task, as well as whether this behavioural response is in conflict or concert with the underlying Pavlovian response. Although the majority of animals are able to learn to avoid a noxious stimulus, many fail to perform at high levels even after training^18^,^19^,^20^. Most studies exclude poor avoiders from analyses due to difficulty in determining whether these animals are suffering from a learning or performance deficiency. This is unfortunate, since these individuals may provide insight into specific neural impairments present in psychiatric disorders involving negative reinforcement deficits, such as addiction and anxiety disorders^21^,^22^. Studies that have examined this subgroup suggest that the breakdown in behaviour does not reflect a learning deficit, but rather one of the performance^18^; these studies reveal extensive freezing during conditioned stimuli that predict shock, which reduces the likelihood the animal will react to avoid punishment.

One way to overcome fear associated with potential shock is to adopt a habitual responding pattern, driven by stimulus–response associations instead of the anticipated negative outcome. This strategy could increase successful avoidance performance during tasks that involve punishment. Indeed, it has been suggested that stress can prompt a transition from goal-directed to habitual responding; specifically, it has been shown that stress makes instrumental responding insensitive to changes in reinforcement value and reduces explicit knowledge of action–outcome contingencies^23^,^24^,^25^,^26^,^27^.

Recent work has begun to address these issues by recording DA release during avoidance-only procedures^7^; however, it is still unclear whether DA correlates seen during avoidance behaviour are similar to those observed during appetitive scenarios. Furthermore, very few studies have examined differences between good and poor avoiders to determine how behaviour and its neural underpinnings vary among individuals^18^,^20^. This information could help explain why some individuals are able to overcome anxiety in stressful situations, while others are not. Here, to address these concerns, we recorded sub-second DA release within the nucleus accumbens core (NAc) using fast-scan cyclic voltammetry as rats performed a combined positive and negative reinforcement procedure. We show that DA release delineates trial type and is higher for both reward and avoidance cues compared with neutral cues only during good avoidance performance, while indiscriminately high DA release is correlated with poor avoidance performance. These results suggest that reward approach and punishment avoidance is signalled within the same microenvironment of the NAc and abnormal processing of these cues may disrupt successful avoidance.

## Results

### Behaviour during combined approach-avoidance

**Figure 1:**
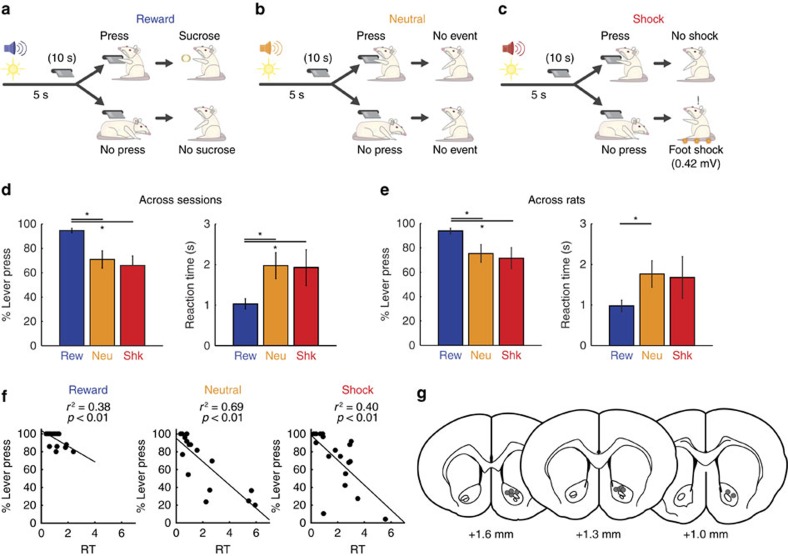
Task design and population behavioural results (*N=*10 rats; 18 sessions). Sessions consisted of 3 trial types: reward (**a**), neutral (**b**), and shock (**c**), which could be identified by their unique auditory cue. (**a**–**c**) At the beginning of each trial, rats were presented with a light cue and trial-specific sound cue 5s before lever extension and then had a maximum of 10 s to press the lever before it was retracted. If rats pressed the lever, they could receive a sucrose pellet reward, avoid an impending foot shock (0.42 mV), or experience no consequence, depending on the identity of the sound cue. If rats failed to press the lever within 10 s after its extension into the chamber, they would alternatively receive no sucrose reward, receive continuous foot shock (0.42 mV), or experience no consequence depending on the identity of the sound cue. Once shock commenced, it could be terminated by lever press. After each consequence, the trial progressed into a 20s ITI. Trial types were pseudo-randomly interleaved within each session (∼60 min) and sound cue identity was counterbalanced across rats. (**d**,**e**) Percent lever press and reaction time computed across each session (**d**) and across rats (**e**). Bars with asterisks represent significance (T-test; *p*<0.05; *n*=18 for (**d**) and *n*=10 for (**e**)). Error bars represent s.e.m. (**f**) Correlation between percentage lever press and reaction time to press for each trial type (reward, neutral, and shock) across all sessions (**g**) Placement of chronic recording electrodes within the NAc core based on histology^62^.

Rats (*n*=10) were trained on a combined approach-avoidance task (Fig. 1a–c). At the start of each trial, one of three discriminatory auditory cues and a cue light were presented. Auditory cues signalled whether the current trial would be a reward, shock or neutral trial. Five seconds after cue presentation, a lever was extended into the chamber where it could be pressed to produce one of three outcomes (dependent on auditory cue identity): delivery of a food reward (positive reinforcement behaviour; reward trials), prevention of foot shock (negative reinforcement behaviour; shock trials), or no consequence (neutral trials). If the animal failed to press the lever within a 10 s period, no food reward was delivered on reward trials, foot shock commenced on shock trials, or there was no consequence on neutral trials. These three trial types were pseudo-randomly interleaved (that is, random without replacement) within each session. The average number of trials per session was 78 (26 per trial type).

The data described below were collected during 18 different behavioural sessions (that is, 3 sessions from 1 rat, 2 sessions per rat from 6 rats and 1 session per rat from 3 rats, to equal 10 rats total) performed in combination with fast-scan cyclic voltammetry (FSCV) recording within the NAc (Fig. 1g). During these sessions, rats produced the most responses and were the fastest to respond on reward trials compared with neutral (% Press (%*P*) value: *t*(17)=3.67, *P*<0.01; reaction time (RT): *t*(17)=3.71, *P*<0.01) and shock trials (%*P* value: *t*(17)=3.88, *P*<0.01; RT: *t*(16)=1.97, *P*=0.07); there was no significant difference between neutral and shock trials for either behavioural measure (%*P* value: *t*(17)=1.42, *P*=0.17; RT: *t*(16)=0.33, *P*=0.74; Fig. 1d). During sessions where at least one shock was delivered (that is, rat failed to avoid shock on at least one shock trial; 15 out of 18 sessions), rats escaped shock on 56% of non-avoid trials. Finally, there was a significant negative correlation between reaction time and per cent lever press for all trial types when examining data across sessions (Reward *r*^*2*^=0.38, Neutral *r*^*2*^=0.69, Shock *r*^*2*^=0.40, all *P<*0.01; Fig. 1f).

Similar results were obtained when we averaged across sessions within a rat and then averaged across rats (that is, one data point for each rat; *n*=10; Fig. 1e). Across rats, percent lever pressing was higher for reward trials relative to neutral (%*P* value: *t*(9)=2.52, *P*<0.05) and shock trials (%*P* value: *t*(9)=2.46, *P*<0.05); there was no difference in lever pressing between neutral and shock trials (%*P* value: *t*(9)=0.88; *P*=0.40). Rats were also slower to respond on neutral and shock trials relative to reward trials; however, this comparison was only significant for neutral versus reward (Rew versus Neu: *t*(9)=2.86, *P*<0.05; Rew versus Shk: *t*(9)=1.22, *P*=0.25). There was no significant difference in reaction times between neutral and shock trials (Neu versus Shk: *t*(9)=0.18, *P*=0.86).

Overall, these behavioural measures demonstrate that rats indeed dissociated reward from the other two trial types; variability in behavioural performance observed across recording sessions will be discussed below. Furthermore, we will show that rats also understood the difference between neutral and shock trials, as illustrated by significantly increased freezing behaviour during the presentation of the shock cue relative to cues predicting reward or neutral trials.

### Phasic DA release is high for approach and avoidance cues

**Figure 2:**
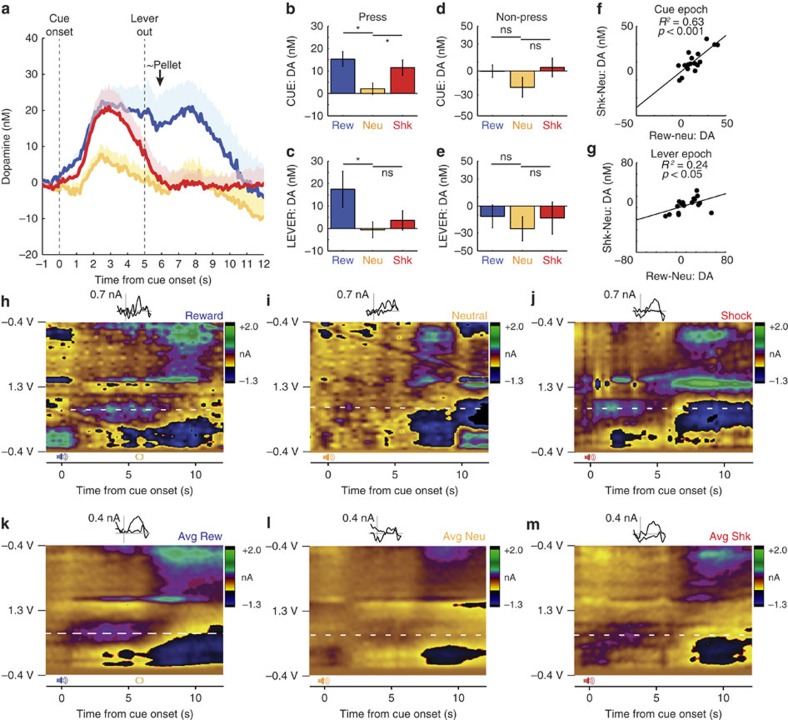
Average dopamine release (*N*=10 rats) during cue and lever epochs for each trial type. (**a**) Dopamine release (nM) across time for reward (blue), neutral (yellow), and shock (red) trials. Dopamine release is baseline (5s before light onset to light onset) subtracted. (**b**–**e**) Quantification of DA release for press and non-press responses during the cue epoch (cue onset to lever extension; 5s) and lever epoch (lever extension plus 1s). Bars with asterisks represent significance (T-test; *P*<0.05). Error bars represent s.e.m. (**f**,**g**) Correlation between shock and reward trials normalized over neutral trials (shock minus neutral; reward minus neutral) for both cue epoch and lever epoch. (**h**–**m**) False-color plots indicate voltammetric current (*z*-axis) plotted against applied scan potential (*y*-axis) and time (*x*-axis) for representative press trials aligned to cue onset for each of the three trial types (**h**–**j**; Reward, Neutral, and Shock), as well as averaged press trials aligned to cue onset for each of the three trial types (**k**–**m**; Average Reward, Average Neutral, Average Shock). Insets show cyclic voltammogram for dopamine; scale bars are set to 0.7 nA for individual examples and 0.4 nA for averages.

As a first step in understanding the role of DA in task performance, we examined changes in phasic DA release across all animals (*n=*10) when rats pressed (‘Press'; Fig. 2a–c) or did not press (‘Non-Press'; Fig. 2d,e) the lever. Average DA release across time is displayed for each of the three trial types in Fig. 2a. Increases in DA release were observed shortly after cue onset and were higher for reward (blue) and shock (red) cues compared to neutral (yellow).

We focused our following analyses on two behaviourally-relevant epochs, a cue epoch (5 s after cue onset) and a lever epoch (1 s after lever extension). Both analysis epochs precede shock and reward delivery, and all data shown are taken before shock delivery to exclude shock artifact. A one-factor analysis of variance (ANOVA) during the cue epoch revealed a significant main effect of trial type during lever press trials (F(2,27)=4.3, *P*<0.05; *n*=10). Mean DA release during both reward and shock cues was significantly elevated compared to neutral trials when rats pressed the lever (Fig. 2a,b; Rew versus Neu: *t*(9)=3.96, *P*<0.01; Shk versus Neu: *t*(9)=2.57, *P*<0.05; *n*=10). DA release during the cue epoch was not significantly different between reward and shock trials (Rew versus Shk: *t*(9)=1.85, *P*=0.10; *n*=10). During the lever epoch, the main effect of ‘trial type' was not significant on lever press trials (F(2,27)=2.56, *P*=0.096; *n*=10); DA release was only significantly elevated during the lever epoch of reward trials, relative to shock and neutral (Fig. 2a,c; Rew versus Neu: *t*(9)=2.75, *P*<0.05; Rew versus Shk: *t*(9)=2.83, *P*<0.05; Shk versus Neu: *t*(9)=0.98, *P*=0.35; *n*=10). False-color plots shown in Fig. 2 indicate voltammetric current (*z*-axis) plotted against applied scan potential (*y*-axis) and time (*x*-axis) for representative press trials aligned to cue onset for each of the 3 trial types (Fig. 2h–j; Reward, Neutral and Shock), as well as averaged press trials aligned to cue onset for each of the three trial types for one session (Fig. 2k–m; Average Reward, Average Neutral, Average Shock). Additional examples of stimulated and behaviourally evoked DA release can be found within the Supplementary Materials (Supplementary Figs 1 and 2). We conclude that, on press trials, DA release was significantly increased for reward and shock trials compared with neutral trials during the cue epoch, but it was only significantly increased for reward trials during the lever epoch. Notably, when rats did not press the lever, there was not a significant main effect of ‘trial type' for either epoch (Cue Epoch: F(2,18)=0.93, *P*=0.41; Lever Epoch: F(2,18)=0.15, *P*=0.86). DA release did not significantly differ between any of the trial types during the cue epoch (Fig. 2d; Rew versus Neu: *t*(4)=2.60, *P*=0.06, *n*=5 and Rew versus Shk: *t*(4)=1.35, *P*=0.25, *n*=5; Shk versus Neu: *t*(7)=1.09, *P*=0.31, *n*=8) or the lever epoch (Fig. 2e; Rew versus Neu: *t*(4)=1.97, *P*=0.12, *n*=5 and Rew versus Neu: *t*(4)=0.68, *P*=0.53, *n*=5; Shk versus Neu: *t*(7)=0.38, *P*=0.72, *n*=8). Note that the degrees of freedom were fewer for the analysis of ‘non-press trials' due to sessions where rats pressed for all trials within a trial type (that is, two rats pressed for all trials across all trial types and two rats pressed for all reward trials but failed to press for some neutral and shock trials).

We see increases in NAc DA during cues that predict potential reward or shock during successful acquisition or avoidance behaviour, respectively. Since this data is averaged across all sessions, it is possible that these increases in DA release to reward and shock cues may have occurred in different microdomains^28^. That is, DA release might be high during reward cues and low during shock and neutral cues in some sessions but high during shock cues and low during reward and neutral cues in other sessions. To address this issue, we computed a reward index (reward−neutral/reward+neutral) and a shock index (shock−neutral/shock+neutral) for each session during the cue and lever epochs. We found significant positive correlations between DA release on reward and shock trials relative to neutral trials during the cue epoch and lever epoch, indicating that increases in DA release to reward cues occurred in the same session and, hence, the same microdomain as increases in DA release to shock cues during avoidance trials (Fig. 2f-g; *r*^2^=0.63 and *r*^2^=0.24, respectively; *P<*0.05 for both; *n*=10 rats).

### DA release is negatively correlated with avoidance

**Figure 3:**
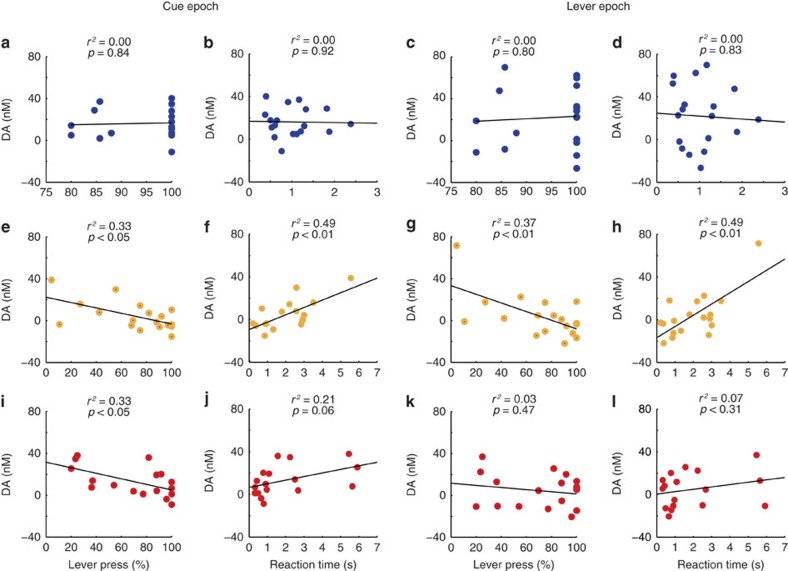
Correlation of DA release with behavioural measures. Each dot represents an individual session; all recording sessions are represented. (**a**–**d**) DA release is not significantly correlated with lever press or reaction time for reward trials (blue). (**e**,**f, i**,**j**) DA release is negatively correlated with lever press and positively correlated with reaction time for both neutral (yellow) and shock (red) trials during the cue epoch. (**g**,**h**) DA release is negatively correlated with lever press and positively correlated with reaction time for neutral trials in the lever epoch. (**k**,**l**) DA is not significantly correlated with behavioural measures for shock trials during the lever epoch.

Next, we examined the relationship between DA release and behaviour during both cue and lever epochs separately for each trial type by plotting percent lever press and reaction time against DA release for all sessions (Fig. 3). For reward trials, correlations were not significant, suggesting that increased dopamine release during the cue or lever epoch does not predict performance or there was not enough variance to capture the relationship between the two (Fig. 3a–d). However, when DA release was high during the cue or lever epoch for neutral trials, reaction times tended to be slower (Cue: *r*^2^=0.491, *P*<0.01; Lever: *r*^2^=0.487, *P*<0. 01; *n*=10 rats) and there were fewer responses on the lever (Cue: *r*^2^=0.333, *P*<0.05; Lever: *r*^2^=0.366, *P*<0.01; *n*=10 rats; Fig. 3e–h). This pattern was conserved for shock trials, but only significant during the cue epoch (%*P* value: *r*^2^=0.327, *P*<0.05; RT: *r*^2^=0.213, *P*=0.06; *n*=10 rats; Fig. 3i–l). Thus, increased DA release during the shock cue was positively correlated with worse performance on the task. This is an intriguing finding, since prior studies predict increased DA during the cue or lever epoch results in more and faster lever pressing for both reward and avoidable shock^7^. Instead, here we find excessive DA at the cue is associated with poor performance during shock avoidance.

### Distinct DA patterns for good and poor avoidance behaviour

**Figure 4:**
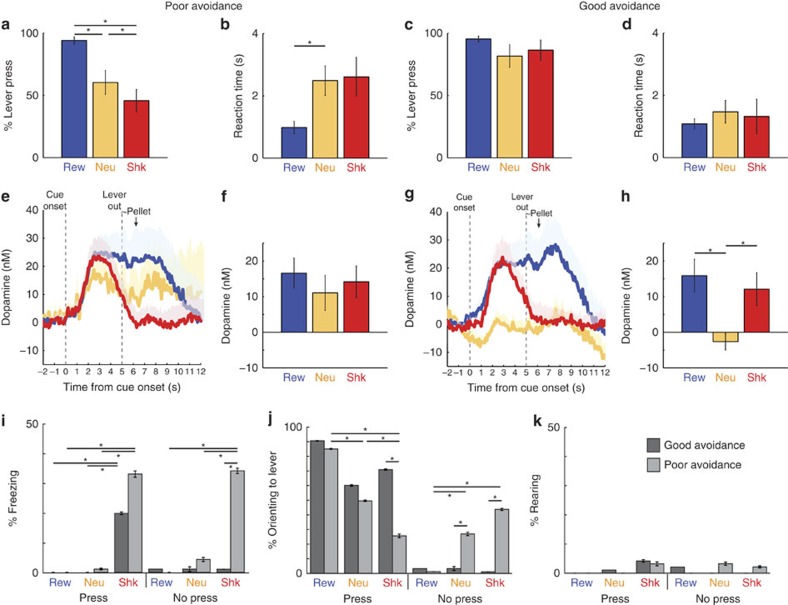
Poor and good avoidance exhibit differences in behaviour and dopamine release across trial types. Reward, neutral, and shock trial types are represented by blue, yellow, and red, respectively. Behavioural differences are shown using percent lever press (**a**) and reaction times (**b**) for poor avoidance and percent lever press (**c**) and reaction times (**d**) for good avoiders. Bars with asterisks represent significance (*T*-test; *P*<0.05; *n*=6 rats for good avoidance, 5 rats for poor avoidance). Error bars represent s.e.m. (**e**–**h**) Dopamine quantification for poor and good avoiders. Dopamine release (nM) for each trial type is shown across time with cue and lever epochs indicated for poor (**e**) and good avoiders (**g**), respectively. Dopamine release is quantified during the cue epoch for each trial type for poor (**f**) and good (**h**) avoiders. Error bars represent s.e.m. (**i**–**k**) Analysis of stress-related behaviours during press and failed press. Percent freezing (**i**), orienting to the lever (**j**), and rearing (**k**) during poor and good avoidance. Asterisks indicate *P*<0.05 in chi-square; *n*=6 rats.

When rats are anxious, they tend to perform poorly in active shock avoidance paradigms due to the perseveration of freezing behaviour, which inhibits the initiation of voluntary actions needed to avoid shock^11^,^20^. In contrast, other rats are able to overcome this Pavlovian response to avoid shock successfully. Based on these findings, we predicted that some rats would press the lever less frequently during cues that predict shock compared to cues that predict neutral trials. As in previous studies, this would enable us to divide our sessions into those displaying good or poor avoidance performance^18^. Indeed, we found a subset of sessions (*n*=9) contained pressing behaviour that differed significantly on shock trials compared with neutral trials. Lever pressing during these sessions showed a significant main effect of trial type in a one-way ANOVA (F(2,24)=8.91, *P*<0.01; *n*=5 rats). During these sessions, response rates were significantly higher and lower for reward and shock trials, respectively, relative to neutral trials (Rew versus Neu: *t*(8)=3.65, *P*<0.01; Rew versus Shk: *t*(8)=5.28 , *P*<0.001; Shk versus Neu: *t*(8)=3.04, *P*<0.05; *n*=5 rats), and reaction times were slower for neutral and shock trials relative to reward trials (Rew versus Neu: *t*(8)=4.23, *P*<0.01; Rew versus Shk: *t*(8)=2.35, *P*=0.05; *n*=5 rats; Fig. 4a,b). Thus, in these sessions, rats pressed significantly less on shock trials compared with reward and neutral trials. We will refer to these sessions as poor avoidance sessions.

The remainder of sessions (*n*=9) showed no significant main effect of trial type on lever pressing (F(2,24)=0.55, *P*=0.58) or reaction time (F(2,24)=0.26, *P*=0.77). Instead, during these sessions, rats pressed at a high rate for all trial types (Rew versus Neu: *t*(8)=1.42, *P*=0.19; Rew versus Shk: *t*(8)=0.63; *P*=0.55; Neu versus Shk: *t*(8)=1.80, *P*=0.11; *n*=6 rats) and were equally fast on neutral and shock trials as reward trials (Rew versus Neu; *t*(8)=1.37, *P*=0.21; Rew vs Shk: *t*(8)=0.64, *P*=0.54; *n*=6 rats; Fig. 4c,d). We will refer to these sessions as good avoidance sessions. When demonstrating good avoidance, rats only received shock on 4.6% of total trials (that is, the sum of all 3 trial types), which was significantly less than 19% received during poor avoidance sessions (*t*(16)= 3.14, *P*<0.01; *n*=6 rats). There was no significant difference between the number of rewards received between groups; during good and poor avoidance sessions rats received reward on 32% and 31% of the total trials (that is, the sum of all three trial types), respectively (*t*(16)=0.86, *P*=0.40; *n*=6 rats).

Thus, overall, six different rats contributed to sessions demonstrating good avoidance (*n=*9 sessions: two sessions per rat for three rats and one session per rat for three rats) and five rats contributed sessions demonstrating poor avoidance (*n=*9 sessions: two sessions per rat for four rats and one session from one rat). Note, only 1 of the 10 recorded rats contributed sessions to both categories (1 and 2 sessions to good and poor avoidance, respectively).

As suggested above, poor avoidance behaviour is thought to result from unmanaged fear-evoked defensive reactions. To determine whether this holds true for our data set, we asked if freezing, lever orienting, and rearing behaviours were different between good and poor avoiders (Fig. 4i–k). Though both groups exhibited increased freezing behaviour during shock trials, poor avoiders froze more than good avoiders (Fig. 4i). Good avoiders (Rew versus Shk: *χ*^2^=15.31, *P*<0.001, Shk versus Neu: *χ*^2^=15.31, *P<*0.001; *n*=3 rats) and poor avoiders (Rew versus Shk: *χ*^2^=24.89, *P*<0.0001, Shk versus Neu: *χ*^2^=22.36, *P*<0.0001; *n*=3 rats) exhibited increased freezing behaviour during shock trials when the lever was pressed, compared with freezing during reward or neutral trials. Freezing on shock trials when rats failed to press the lever was significantly increased relative to reward and neutral trials (Shk versus Rew: *χ*^2^=25.66, *P*<0.0001; Shk versus Neu: *χ*^2^=16.78, *P*<0.0001; *n*=6 rats) and relative to shock trials when good avoiders did not press the lever (Poor Shk Non-press versus Good Shk Non-press: *χ*^2^=23.91, *P*<0.0001; *n*=6 rats). Thus, we found that poor avoiders froze more on both press and non-press shock trials when compared with good avoiders. Notably, good avoiders still expressed fear responses during shock trials, demonstrating that they clearly understood task contingencies.

Rats generally oriented towards the lever more often when they were successful in pressing (Fig. 4j). Poor avoiders showed significant step-wise decreases in orienting behaviour following the same pattern as their lever pressing behaviour (Rew versus Neu: *χ*^2^=4.75, *P*<0.05; Rew versus Shk: *χ*^2^=18.91, *P*<0.0001; Shk versus Neu: *χ*^2^=4.61, *P*<0.05; *n*=3 rats); orienting behaviour was not significantly different between trial types when good avoiders pressed the lever (Rew versus Neu: *χ*^2^=2.98, *P*=0.08; *n*=3 rats). Poor avoiders oriented toward the lever more often during failed shock trials and neutral trials than during failed reward trials (Rew versus Neu: *χ*^2^=29.85, *P*<0.0001; Rew versus Shk: *χ*^2^=17.86, *P*<0.0001; *n*=3 rats), unlike good avoiders who failed to orient on non-press trials regardless of trial type (Good versus Poor for Neu: *χ*^2^=14.02, *P*<0.001; Good versus Poor for Shk: *χ*^2^=30.82, *P*<0.0001; *n*=6 rats). There were no significant differences in rearing behaviour, a measure of general motor activity, attention, and environmental engagement^29^, between good and poor avoiders across any trial type (Fig. 4k).

To determine how DA release patterns differ among good and poor avoidance behaviours, we performed a three-factor ANOVA across trial type (reward, shock and neutral), group (good or poor avoidance), and response type (press or non-press). This revealed a main effect of response type (F(1,75)=7.07, *P*<0.01; *n=*10), trial type (F(2,75)=5.83, *P*<0.01; *n=*10), and group (F(1,75)=6.92, *P*<0.05; *n=*10). In addition, there was a significant two-way interaction between trial type and group (F(2,75)= 3.38, *P*<0.05; *n=*10). Interactions between response type and group (F(1,75)=1.29, *P*=0.28; *n=*10), between response type and trial type (F(2,75)= 0.77, *P*=0.47; *n=*10), and between all three factors did not achieve significance (F(2,75)=2.49, *P*=0.09; *n=*10).

Next, we examined average DA release over time for good and poor avoidance sessions (Fig. 4e–h). When rats performed poorly on avoidance trials, DA release was nonselective during the cue epoch (Fig. 4f); there was no main effect of trial type in the one-factor ANOVA (F(2,23)=0.33, *P*=0.72; *n*=5 rats) and no comparisons between trial types were significant (Rew versus Neu: *t*(8)=1.22, *P*=0.25; Rew versus Shk *t*(7)=1.04, *P*=0.33; Shk versus Neu: *t*(7)=2.09, *P*=0.074; *n*=5 rats). To the contrary, when rats that responded at a high rate for all trial-types (that is, demonstrating good avoidance), DA release clearly delineated reward, shock, or neutral cues (Fig. 4g). During the cue epoch, we found a significant main effect of trial type (Fig. 4h; F(2,24)=5.37, *P*<0.05; *n*=6 rats) and DA release during both reward and shock cues differed from release seen during neutral cues (Rew versus Neu: *t*(8)=3.81, *P*<0.01; Shk versus Neu: *t*(8)=3.01, *P*<0.05; Rew versus Shk: *t*(8)=1.27; *P*=0.24; *n*=6 rats; Fig. 4h).

With these group distinctions in mind, we re-examined the correlation between DA release and behaviour. During good avoidance, DA release was not correlated with behaviour (% P or RT) for any trial type or analysis epoch (Supplementary Table 1). However, during poor avoidance, DA release was negatively correlated with % P during both neutral (Cue: *r*^*2*^=0.60, *P*<0.05; Lever: *r*^*2*^=0.66, *P*<0.01; *n*=5 rats) and shock trials (Cue: *r*^*2*^=0.66, *P*<0.05; *n*=5 rats; Supplementary Table 1). In these sessions, DA release was also positively correlated with reaction times during both neutral (Cue: *r*^*2*^=0.62, *P*<0.05; Lever: *r*^*2*^=0.76, *P*<0.01; *n*=5 rats) and shock trials (Lever: *r*^*2*^=0.58, *P*<0.05; *n*=5 rats). Altogether, these data suggest that increased cue-evoked dopamine release in poor avoiders promotes maladaptive behaviour such as slower and fewer lever presses during avoidance, but not during reward-seeking.

## Discussion

While dopaminergic activity within the mesolimbic pathway has been widely implicated in the construction of reward expectations, a growing literature has recently emerged investigating its role during punishment and avoidance. Recent studies suggest increased cue-evoked DA release in the NAc predicts punishment avoidance, whereas a pause in DA transients occurs during unavoidable punishment across modalities^7^,^30^,^31^,^32^,^33^,^34^. Yet, activation of DA neurons and D1 receptors is necessary for the formation of fear memories, and increases in DA release in the NAc core occurs in direct response to punishments, such as tail pinch^35^,^36^,^37^. These seemingly contradictory findings have made it difficult to pinpoint the exact role of DA during punishment and negative reinforcement.

Here we show that phasic increases in DA release can signal the need for approach or avoidance behaviour within the same microenvironment. Our group data reveal higher cue-evoked DA release during shock and reward cues compared with neutral cues, when the cue promotes lever press. By temporally dissociating the onset of the cue and the extension of the lever, we also found that the increase in DA release seen during shock avoidance is to the cue, not the action. Importantly, increased DA release to cues predicting shock and reward do not appear to reflect salience, since cues that predict unavoidable shock—although salient—inhibit DA release^7^,^38^,^39^. Taken together, these results suggest that increased DA release to cues predicting successful avoidance and reward-seeking report the predicted value associated with each.

Our results are consistent with a previous report from Oleson et al. showing increased DA release to cues that predict successful avoidance^7^; however, their study also found increases in DA release during a cued safety period, when shock would have been delivered had the animal not successfully pressed the lever to avoid it. This increase in DA release was interpreted as a reinforcement signal similar to those seen during reward delivery in appetitive tasks. It is worth noting that our current study did not overtly signal entry into the safety period, and, in turn, we did not witness an increase in DA release during this time point in our data set. There are several possible explanations as to why we did not replicate this effect. First, Oleson *et al*. presented a safety cue that turned on after rats successfully avoided foot shock. In our task, there was no cue to explicitly signal safety from shock. It is possible, then, that an external safety cue is necessary to elicit a DA response during the safety period and these increases will not occur simply to the absence of predicted shock. Secondly, our rats may have been more thoroughly trained in our task than rats were in the previous report, and, thus, DA release could have completely transferred to the avoidance cue; however, this was not the case for reward trials. Lastly, not getting shocked when a potential shock was predicted is an outcome that is better than expected; this is true in both behavioural paradigms. However, in our task, the shock trial type also implicitly signifies that food reward will not be delivered. It is possible that any increases in DA release we would have seen during the safety period were attenuated by a simultaneous pause in DA release that occurs in the absence of a food reward. Further research will be necessary to rule out these interpretations; however, it was clear in both studies that DA release was high during cues that predicted successful shock avoidance.

We only observed increases in DA release during reward and shock cues relative to neutral cues when rats demonstrated good avoidance behaviour. These animals responded reliably and at comparably high speeds for all three predictive cues. Compared to poor avoiders, good avoiders also froze less to cues predicting shock and responded quickly on shock trials. Thus, this group seems to be responding without being deterred by the potential negative outcome of shock trials, as if they were responding habitually. The development of a habit-like strategy is supported by previous research showing that stress can lead to an insensitivity to changes in reinforcement value and a reduction in explicit knowledge of action-outcome contingencies^23^,^24^,^25^,^26^,^27^. Both goal-directed and habitual processes are thought to be involved in successful avoidance learning, and the behavioural pattern of good avoiders could reflect the utilization of a proactive habitual strategy under the control of dorsal lateral striatum (habit center) to maximally obtain reward and avoid punishment^40^. However, note that in our task this remains speculation, since our current data set does not allow us to prove that our rats were acting habitually in response to all three trial type cues. Recent work has shown that rats well-trained on avoidance paradigms still show sensitivity to the devaluation of the shock outcome, which suggests that they remain goal-directed with respect to this action–outcome contingency^41^. This could suggest, then, that the NAc is monitoring predictions but does not directly initiate action in this task unless there are changes in action-outcome contingencies. Indeed, we found that DA release during good avoidance was not correlated with behavioural output; despite this, DA release clearly and correctly reflected the value of the predictive cues. Such signals are likely critical to maintaining appropriate responding behaviour during our task, consistent with previous studies demonstrating that NAc lesions (6-hydroxydopamine, quinolinic acid and electrolytic) and D1 receptor antagonists disrupt avoidance behaviour^42^,^43^,^44^,^45^.

On the basis of the existing literature, it would be logical to conclude that poor avoidance behaviour likely reflects low phasic DA release in NAc to shock cues. However, with few exceptions^18^,^20^, current animal research on avoidance behaviour has focused on subjects who avoid at high rates. Animals that perform poorly on avoidance tasks are often omitted under the assumption that they fail to learn task contingencies; however, it has been shown that poor avoiders do learn and instead suffer from performance deficits that arise from persistent species-specific defense reactions^11^,^18^,^20^,^46^,^47^. For example, poor avoiders tend to demonstrate higher baseline levels of anxiety and exhibit persistent freezing behaviour^20^,^46^,^47^. For these reasons, poor avoiders might better represent human populations with psychiatric disease.

During poor avoidance sessions in our task, when rats responded most for reward and least for shock trials, DA release during the cue was indiscriminately high across all trial types. Thus, DA release failed to properly reflect the value of cues, including cues predicting failed shock avoidance and neutral trials, when an animal's behaviour was ruled by the fear of an expected aversive outcome. Such a signal could confuse processing in downstream areas, where the predictive value of future action or inaction would be indistinguishable. Increased lever pressing during reward trials versus neutral or shock trials might reflect higher overall value associated with the combined promise of reward and relief of avoiding shock; however, we do not feel that this is a complete explanation, since rats do not press more for reward than shock during good avoidance and rats also press more for neutral than shock during poor avoidance. We also found that decreased responding on shock versus neutral trials corresponded with increases in freezing to the cue, reflecting a species-specific defense reaction described previously^11^,^18^,^20^; high DA release preceding failed avoidance might also reinforce these inappropriate freezing behaviours during avoidance trials. Recent studies have suggested that misappropriated increases in DA release to irrelevant or misinterpreted stimuli, like our neutral cues or failed shock avoidance cues, could be critically linked to dysfunctional salience attribution in many psychological disorders^48^,^49^,^50^,^51^,^52^,^53^. In contrast, accumbal DA release during good avoidance clearly assigned value to cues based on their predictive valence, namely exhibiting high DA release for lever press trials during which reward was obtained or punishment was avoided.

Altogether, these data suggest that abnormal processing of value signals in NAc hinders adaptive behaviour during active avoidance. That is, when rats are intractably focused on the outcome, avoidance performance is poor and is correlated with higher overall DA release in NAc. Though reliance on expected outcomes is adaptive for behaviour driven by rewards and their predictive cues, this is maladaptive during punishment avoidance. These results should provide insight into the underlying neural mechanisms involved in psychiatric disorders such as addiction, anxiety disorders, and psychosis.

## Methods

### Animals

Sixteen male Sprague-Dawley rats were obtained from Charles River Labs at 300–350 g (90-120 days old). Animals were individually-housed in a temperature- and humidity-controlled environment and kept on a 12-h light/dark cycle (07:00–19:00 in light); all tests were run during the light phase. Animals had access to water *ad libitum* and body weight was maintained at 85% of baseline weight by food restriction (15 g standard rat chow provided daily, in addition to ∼1 g sucrose pellets during experimental trials). Of the 16 animals entering the study, 10 animals provided reliable cyclic voltammograms. All procedures were performed in concordance with the University of Maryland, College Park Institutional Animal Care and Use Committee (IACUC) protocols.

### Chronic microelectrode fabrication

Electrodes were constructed according to the methods of Clark *et al*.^54^. A single carbon fiber (Goodfellow Corporation) was inserted into a 15 mm cut segment of fused silica (Polymicro Technologies) while submerged in isopropyl alcohol. One end of the silica tubing was sealed with a two-part epoxy (T-QS12 Epoxy, Super Glue) and left to dry overnight, leaving untouched carbon fiber extending past the seal. The protruding carbon fiber was cut to a length of 150 μm. A silver connector (Newark) was secured to the carbon fiber at the opposing end of the silica tubing using silver epoxy (MG Chemicals) and was allowed to dry. A final coat of two-part epoxy was then applied to the pin connection to provide insulation and structural support for the electrode and was allowed to dry overnight.

### Intra-cranial surgical procedures

All animals were anaesthetized using isoflurane in O_2_ (5% induction, 1% maintenance) and implanted with a chronic voltammetry microelectrode aimed at the NAc core (+1.3 AP, +1.4 ML, −6.9 DV), an ipsilateral bipolar stimulating electrode (Plastics One) in the medial forebrain bundle (−2.8 AP, +1.7 ML, −8.8 DV), and a contralateral Ag/AgCl reference electrode (Sigma-Aldrich). The reference electrode and anchoring screws were stabilized using a thin layer of dental cement (Dentsply), leaving the holes for the stimulating and recording electrodes unobstructed. The stimulating and recording electrodes were attached to a constant current isolator (A-M Systems) and voltammetric amplifier, respectively, and lowered to the most dorsal point of the target region (–6.6 DV for the working electrode and −8.5 DV for the stimulating electrode). At this depth, a triangular voltammetric input waveform (−0.4 to +1.3−V versus Ag/AgCl, 400 V/s; Heien *et al*.^55^) was applied to the recording electrode at 60 Hz for 30 min and then reduced to 10 Hz for the remainder of the surgery. Electrical stimulation (24 biphasic pulses, 60 Hz, 120 μA) was applied to the stimulating electrode to evoke dopamine release, which was monitored at increasing depths by the recording electrode. If neither an evoked change in DA nor a physical response (whisker movement or blinking) was observed, the stimulating electrode was lowered by 0.05 mm until a response was achieved or to a maximum depth of 8.8 mm. The working electrode was then lowered by 0.05mm until DA release was observed or to a maximum depth of 6.9 mm. Once electrically-evoked DA release was detected in the NAc core, a thin layer of dental cement was used to secure the stimulating and recording electrodes in place (Supplementary Fig. 3). A Ginder implant (Ginder Scientific; constructed in house) was connected to the reference, stimulating, and recording electrodes and fully insulated using dental cement, leaving only the screw-top connector exposed, to reduce noise and prevent loss of connectivity during behavioural training. Animals then received post-operative care: subcutaneous injection of 5 ml saline containing 0.04 ml carprofen (Rimadyl), topical application of lidocaine cream to the surgical area, and placement on a heating pad until full consciousness was regained. Animals were also given antibiotic treatment with Cephlexin orally twice daily post surgery for 2 weeks to prevent infection of the surgical site. All subjects were allowed a month for full recovery and stabilization of the electrode before experimentation.

### Combined positive and negative reinforcement behavioural task

Animals were first trained daily on a 45 min foot shock (0.42 mA) escape procedure to establish the response-shock termination contingency. Foot shock intensity was selected based on the conditioned foot shock intensity optimization protocol for avoidance behaviour outlined in Oleson *et al*^7^. For behavioural sessions accompanied with FSCV recording, we used the moderately aversive stimulus strength of 0.42 mV to balance aversiveness with response probability; however, our task employed continuous shock for punishment as opposed to intermittently spaced shock, as used in Oleson *et al*^7^. During each session, subjects were presented with a lever paired with a cue light and an auditory cue; a response on the lever at any point during the session resulted in the retraction of the lever and termination of the cue light and foot shock, as well as progression to the ITI (20 s). Subjects were gradually shaped toward the lever (safe side, quadrant with lever, orientation toward the lever, rearing, pressing) by the experimenter as needed until escape behaviour acquisition.

Once subjects acquired consistent escape behaviour, trials were altered to allow for shock avoidance; positive reinforcement and neutral contingencies were also added. At trial onset, a cue light and one of three discriminatory auditory cues (tone, white noise or clicker) were activated; house lights remained on at all times. After 5 s, the lever was extended into the chamber; the 5 s delay was implemented to reduce compulsory pressing and to allow for separate epoch analysis around cue and lever press. Once extended, the lever could be pressed to produce one of three outcomes (dependent on the auditory cue identity): delivery of a food reward (a sucrose pellet; positive reinforcement behaviour), prevention of foot shock (0.42 mV; negative reinforcement behaviour), or no consequence. If the animal failed to press the lever within a 10 s period, no food reward was delivered, foot shock commenced, or no there was no consequence. Similar to the previous protocol, rats were able to press the lever at any time to escape the foot shock once it commenced; if rats failed to press the lever, foot shock automatically terminated after 15 s. After response or termination of the trial, an ITI of (20 s) was initiated. Auditory cue identities were counterbalanced across rats. Animals were very well trained on this task, completing >30 sessions and displaying >60% avoidance responses for three consecutive sessions before recording. Session duration during FSCV recording was 60 min.

### Fast-scan cyclic voltammetry

For recordings, animals were connected to a head-mounted voltammetric amplifier (current-to voltage converter) and a commutator (Crist Instruments) mounted above the recording chamber. During each session, an electrical potential was applied to the recording electrode in the same manner as described above (see Intra-cranial surgical procedures). To detect changes in dopaminergic concentration over time, the current at its peak oxidation potential was plotted for successive voltammetric scans and background signal was subtracted. Two PC-based systems, fitted with PCI multifunction data acquisition cards and software written in LabVIEW (National Instruments), were used for waveform generation, data collection, and analysis. The signal was low-pass filtered at 2,000 Hz. Event timestamps from Med Associates were recorded, to analyse behaviourally relevant changes in dopamine release.

Dopamine was identified by its stereotypical and specific cyclic voltammogram signature. Behaviourally evoked DA signals met electrochemical criterion if the cyclic voltammogram was highly correlated to that of the DA templates produced during the training set. The training set is a template containing six each of background-subtracted cyclic voltammograms and corresponding calibrated concentrations for both dopamine and pH extracted from data pooled across animals acquired during electrical stimulations that are known to evoke DA release (stimulation at 1 V: 30 Hz, 6 pulses; 30 Hz, 12 pulses; 30 Hz, 24 pulses; 60 Hz, 6 pulses; 60 Hz, 12 pulses; 60 Hz, 24 pulses). The data collected during a session were not analysed if reward trials did not elicit DA release that satisfied these chemical verification criteria. Voltammetric data was analysed using software written in LabView and Matlab. A principal component regression (Tar Heel CV chemometrics software) was used to extract the DA component from the raw voltammetric data^56^,^57^. Eigenvalues (principal components) are calculated that describe relevant components of our training set, and we perform multivariate regression analysis to determine a correlation coefficient to describe our recorded behavioural data versus the training set. The number of factors we select to keep in our PCA analysis accounts for >99% of the variance (at least 3, but usually 4–5 factors are kept). Factor selection is a very important step, as retaining more factors than we need would add noise to our data but retaining too few could mean discarding potentially meaningful information^58^. FSCV results may be influenced by the way in which the variance is apportioned to the components. Importantly, the exact same method was applied to each trial-type (neutral, reward, and shock) allowing for fair comparison between conditions.

We also use the residual to examine the quality of the fit. In general, the residual is the difference between the experimental observation and the predicted value derived from a model/template (our regression values) and is a measure of the unknown portion of the signal that is not accounted for by the principal components of the regression. This is important when considering the accuracy and the applicability of the model and is important for identifying possible interfering molecules or noise (such as drift). The sum of squares of the difference between the template and the experimental data is the residual value (Q) and the threshold Qa establishes whether the retained principal components provide a satisfactory description of the experimental data; the discarded principal components should provide a measure of noise^57^,^59^. We use this Qa measure in combination with our regression analysis to establish our concentration corrections.

Chemometrics is a widely used analytical method that separates changes in current that are caused by DA release from those caused by pH shift or other electrochemical ‘noise' by comparing eigenvalues derived from stimulated DA release and changes in pH to those derived from behavioural release^28^,^59^,^60^,^61^.

### Histology

Following the completion of the study, animals were terminally anesthetized with an overdose of isoflurane (5%) and transcardially-perfused with saline and 4% paraformaldehyde. Brain tissue was removed and post-fixed with paraformaldehyde. Brains were then placed in 30% sucrose solution for 72 h and sectioned coronally (50 μm) using a microtome. Tissues slices were mounted onto slides and stained with thionin for histological reconstruction.

### Data analysis and statistics

Behavioural videos from the combined positive and negative reinforcement task were scored for measures of fear (freezing, rearing and orienting to the lever) during the cue presentation epoch (cue onset to lever extension) for all trial types. For behavioural analysis, this epoch was divided into 2 sub-epochs (first half and last half) and separate binary (0 or 1) scores were recorded for each behavioural measure during each sub-epoch. These behavioural analyses were scored blindly.

As described above, all voltammetric data was analysed using software written in LabView and then further analysed in Matlab (Mathworks). The dopamine component of our signal was first isolated from the raw voltammetric signal using principal component regression and calibration to a CV/concentration matrix. Analysis was centered on various epochs: cue epoch (cue onset to lever extension), lever epoch (1 s after lever extension), and baseline epoch (5 s before cue onset). Behavioural measures were correlated to dopamine release using linear regression (*P*<0.05).

### Data availability

All data that support the findings of this study are available from the corresponding author on request.

## Additional information

**How to cite this article:** Gentry, R. N. *et al*. Phasic dopamine release in the rat nucleus accumbens predicts approach and avoidance performance. *Nat. Commun.* **7,** 13154 doi: 10.1038/ncomms13154 (2016).

## Supplementary Material

Supplementary Information Supplementary Figures 1-3 and Supplementary Table 1
