# Supplementary material for: Phasic dopamine release in the rat nucleus accumbens predicts approach and avoidance performance
Source: Nat Commun. 2016 Oct 27;7:13154. doi: 10.1038/ncomms13154 (PMC5095290; doi:10.1038/ncomms13154)
Supplement: Supplementary Information — Supplementary Figures 1-3 and Supplementary Table 1 [file ncomms13154-s1.pdf]

Supplementary Figure 1

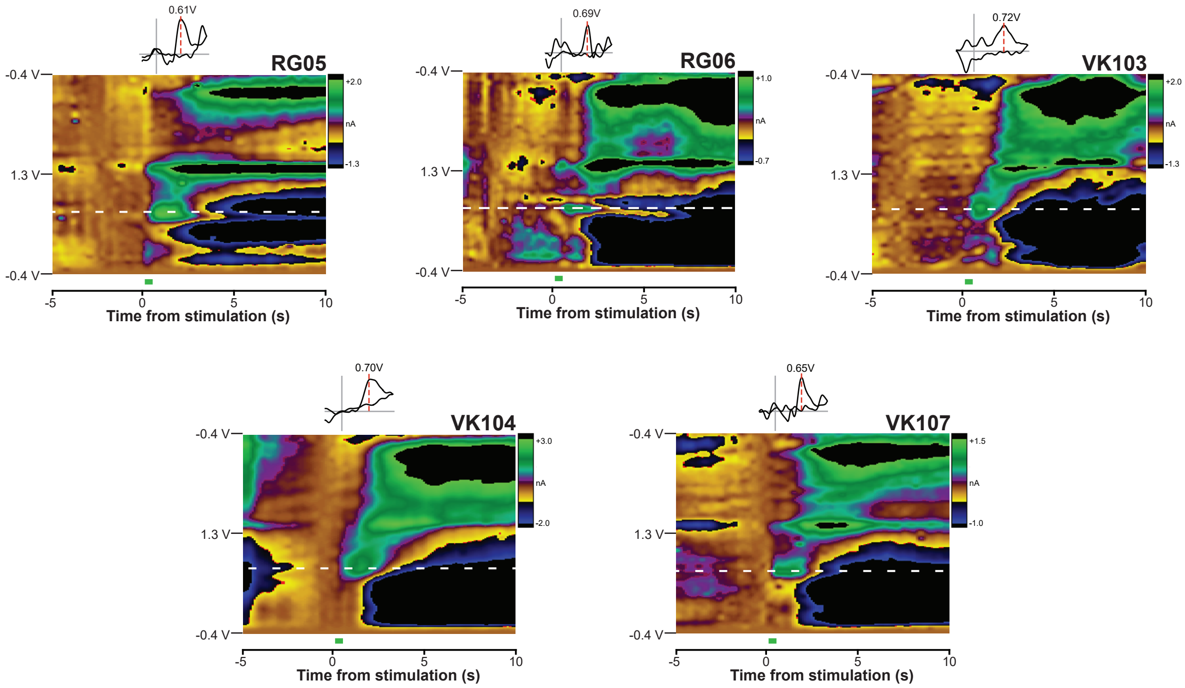

Supplementary Figure 1. Additional false-color examples showing dopamine release during five post-session stimulations. False-color plots indicate voltammetric current (*z*-axis) plotted against applied scan potential (*y*-axis) and time (*x*-axis) for representative press trials aligned to stimulation onset (t = 0). Examples show dopamine release to the strongest stimulation parameter (1V, 24 pulses at 60 Hz) from the training set for each of five animals.

Supplementary Figure 2

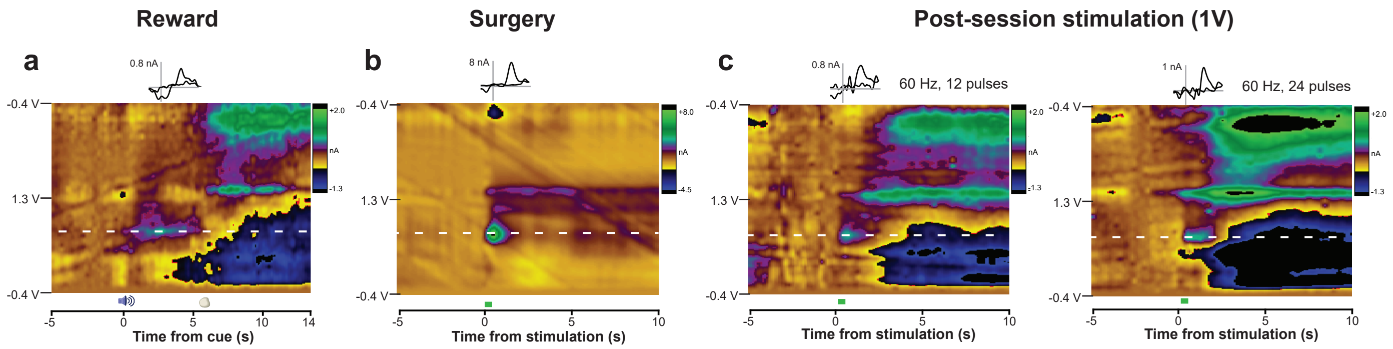

Supplementary Figure 2. Additional false-color examples showing dopamine release. False-color plots indicate voltammetric current (*z*-axis) plotted against applied scan potential (*y*-axis) and time (*x*-axis) for representative press trials aligned to cue onset for reward (**a**), as well as stimulated DA verification trials taken at final depth during surgery (**b**) and during two post-session stimulations from the session training set (**c**).

Supplementary Figure 3

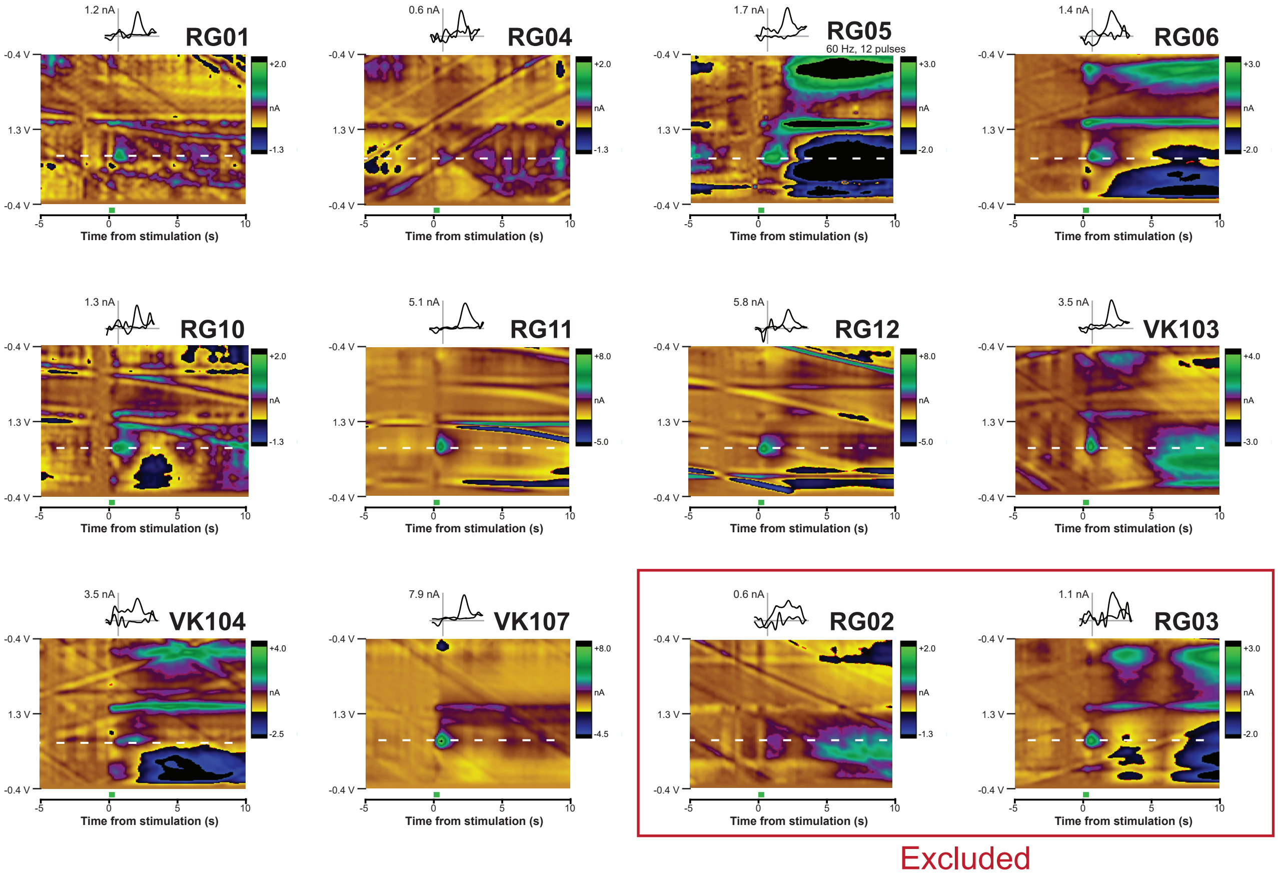

Supplementary Figure 3. False-color examples showing stimulated DA verification trials taken at final depth during surgery for all animals used during recordings. False-color plots indicate voltammetric current (*z*-axis) plotted against applied scan potential (*y*-axis) and time (*x*-axis). Two animals were excluded from analyses due to an unreliable waveform (RG02) and the loss of a headcap (RG03).

Supplementary Table 1

a

Poor Avoiders

		Cue Epoch			Lever Epoch		
		Reward	Neutral	Shock	Reward	Neutral	Shock
PC	R^2	0.25(+)	**0.60(-)**	**0.66(-)**	0.28(+)	**0.66(-)**	0.46(-)
	p	0.1667	**0.0145**	**0.0149**	0.1451	**0.008**	0.0646
RT	R^2	0.08(-)	**0.62(+)**	0.21(+)	0.05(-)	**0.76(+)**	**0.58(+)**
	p	0.4604	**0.0120**	0.2557	0.5515	**0.0023**	**0.0276**

b

Good Avoiders

		Cue Epoch			Lever Epoch		
		Reward	Neutral	Shock	Reward	Neutral	Shock
PC	R^2	0.23(-)	0.00	0.28(-)	0.29(-)	0.00	0.04(+)
	p	0.1889	0.8862	0.1419	0.1350	0.9502	0.5897
RT	R^2	0.07(+)	0.08(+)	0.23(+)	0.03(+)	0.00	0.07(-)
	p	0.4855	0.4490	0.1954	0.6815	0.8589	0.4813

Supplementary Table 1. Correlation between DA release and behavior in poor and good avoiders. R^2 values (top) and *p* values (bottom) for correlations between DA release during each trial type and behavior during cue and lever epochs for poor (**a**) and good (**b**) avoiders.
